# An overview of seventy years of research (1944 – 2014) on toxoplasmosis in Colombia, South America

**DOI:** 10.1186/1756-3305-7-427

**Published:** 2014-09-04

**Authors:** William Alberto Cañón-Franco, Natalia López-Orozco, Jorge Enrique Gómez-Marín, Jitender P Dubey

**Affiliations:** Departamento de Medicina Veterinária Preventiva e Saúde Animal, Faculdade de Medicina Veterinária e Zootecnia, Universidade de São Paulo, Av. Prof. Dr. Orlando Marques de Paiva 87 Cidade Universitária, São Paulo, SP CEP 05508-270 Brasil; Laboratorio de Parasitología Veterinaria, Departamento de Salud Animal, Facultad de Ciencias Agropecuarias, Universidad de Caldas, Calle 65 No. 26-10, Manizales, Colombia; Grupo de Estudio en Parasitología Molecular (GEPAMOL), Centro de Investigaciones Biomédicas, Universidad del Quindío, Av. Bolivar 12 N, Armenia (Quindío), Colombia; United States Department of Agriculture, Agricultural Research Service, Beltsville Agricultural Research Center, Animal Parasitic Diseases Laboratory, Beltsville, MD 20705-2350 USA

**Keywords:** Colombia, Epidemiology, *Toxoplasma gondii*, Toxoplasmosis, Congenital, Ocular, Genotypes, Public health, Outbreaks

## Abstract

This paper summarizes prevalence of *Toxoplasma gondii* in humans and animals and associated correlates of infection, clinical spectrum of disease in humans, and genetic diversity of *T. gondii* isolates from Colombia. Recent studies, especially in the states of Antioquia, Quindío and Cundinamarca, indicate that toxoplasmosis is a major public health problem. Approximately half of the women of child bearing age have *T. gondii* antibodies, and the clinical disease in congenitally infected children is more severe than in Europe. Limited studies indicate that the strains of *T. gondii* from Colombia are genetically and phenotypically different than in Europe and North America. However, epidemiological factors, such as the involvement of domestic and/or wild animals in transmission, the distribution of strain diversity by natural geographic regions, and the variation in risk factors between regions that are associated with human infection in Colombia, remain unknown. Areas of research for the future are outlined. This review should be of interest to biologists, veterinarians, physicians, and parasitologists.

## Review

Toxoplasmosis is a worldwide zoonosis with asymptomatic infections in most adult immunocompetent humans. Why some persons infected with *Toxoplasma gondii* become sick and even die is not completely understood. Recently, in French Guiana, immunocompetent adults died of toxoplasmosis [1]. In Brazil, a higher proportion of congenitally infected children developed severe disease and the onset of clinical symptoms was also earlier than such cases from the rest of the world [2].

Host and/or parasite factors play a pathogenic role. In addition, it has been hypothesized that the strains of *T. gondii* involved might influence the severity of toxoplasmosis [3]. Recent studies indicate that the strains of *T. gondii* from South America are phenotypically and genetically different from those in Europe and North America [4, 5]. Information obtained from studies in Brazil and some recent studies in Colombia indicate that a similar scenario might apply to both countries with severe clinical consequences in congenitally infected children.

Although Colombia has the third greatest human population of South America (47 million habitants) and harbors one of the highest biodiversities in the world [6], there is no systematic review of the literature for studies on toxoplasmosis. Here, we review toxoplasmosis in humans and animals from Colombia and highlight the need for further studies on toxoplasmosis as a real public health problem.

### History and introduction

Historically, *T. gondii* was first found in a Colombia in a naturally infected guinea pig (*Cavia porcellus*) [7] but little else was known until Roca-García *et al.* [8] published the first detailed description of a case of congenital toxoplasmosis in a 40 day-old girl born to an apparently healthy mother from Bogota, Colombia. The child had classical Sabin’s tetrad symptoms of congenital toxoplasmosis including bilateral chorioretinitis, hydrocephalus, cerebral calcifications, jaundice, hepatomegaly and splenomegaly. *T. gondii* was isolated from the cerebrospinal fluid by bioassay in mice and the strain was found to be virulent for mice, guinea pigs, rabbits, chickens and pigeons. These authors provided an update of toxoplasmosis in children worldwide at that time [8].

The need for prenatal screening of women was recognized in 1970’s, and Restrepo *et al.* [9] made an initial attempt to screen 120 women. They found that half of the women were seronegative and 8.3% became seropositive during pregnancy; viable *T. gondii* was isolated from 10 of 30 placentas obtained from these women [9]. These observations eventually led to the first national serological study in the general population, in 1980, and then to a multicenter study of congenital toxoplasmosis [10].

Varela and Roca [11] performed the first serological survey in Indian Guambias in Cauca, Colombia by using the Sabin Feldman dye test (SF). Feldman [12], coauthor of the SF test, reported that *T. gondii* seroprevalence in Colombia was approximately four times that in the USA. He tested sera from military recruits and found that seroprevalence was 50% of 2,803 from Colombia, 56% of 2,023 from Brazil, and 14% 2,680 from the USA using the SF test. These findings are of historic importance because we are not aware of any other study where prevalence has been compared in humans (all males) of one age group from three countries by one laboratory using identical methodology.

The SF dye test is the most specific test for the detection of antibodies to *T. gondii* in humans and even low titers (1:4–1:16) are considered specific [13]. However, this test is technically difficult, hazardous to perform (because live virulent *T. gondii* are needed for the test), and not used now in Colombia or in most other countries. Since then, several other serological tests have been developed, and data based from other tests are not always comparable. We have summarized in Table 1 all serological tests used for studies in Colombia, so that readers can draw their own conclusions concerning the prevalence of *T. gondii* antibodies in humans and animals summarized throughout the review. Cut-off values are indicated in Tables wherever the authors provided the information.

**Table 1:** **Technical features of serological tests used for detection of** ***T. gondii*** **antibodies in humans and animals in Colombia**

Test, abbreviation	Antigens	Manufacturer	Tables referred
**Sabin Feldman dye test, SF**	Live tachyzoites	In-house	2, 3
**Skin test**	Soluble	In-house	2, 4
**Indirect haemagglutination, IHAT**	Soluble	Not stated	5
**Indirect haemagglutination, IHAT**	Soluble	Behringwerke, Germany (Note, these company have merged into CSL Behring) http://www.cslbehring.com	5
**Indirect haemagglutination, IHAT**	Soluble	Not state	2
**Indirect fluorescent antibody, IFAT**	Inactivated	Not stated	2, 3, 5
**Indirect fluorescent antibody, IFAT**	Inactivated	Cappel Laboratories, Cochranville PA, USA	5
**Indirect fluorescent antibody, IFAT**	Inactivated	CSL Behring, Germany http://www.cslbehring.com	2, 3
**Indirect fluorescent antibody, IFAT**	Whole formaldehyde- fixed tachyzoites	National Institute of Health, Santa fé de Bogotá, Colombia http://www.ins.gov.co	2, 3, 4, 5
**Modified Agglutination, MAT**	Formalin-treated whole tachyzoites	Biomérieux, Craponne, France http://www.biomerieux.com	5
**Immunosorbent Agglutination Assay test, ISAGA**	Whole tachyzoites-killed	Biomérieux, Craponne, France http://www.biomerieux.com	2
**Enzyme linked fluorescent assay, ELFA** VIDAS® EBV IgG, IgM	Soluble,	Biomérieux, Craponne, France http://www.biomerieux.com	2, 3, 4
**Enzyme linked immunosorbent assay, ELISA**			
1. Micro ELISA	Soluble	Not stated	2, 4
2. TOXO IgG Detect ELISA Kit	Inactivated	BioKit, Barcelona, Spain http://www.biokit.com	2, 4
3. *Toxoplasma* ELISA IgG, IgM	Soluble	Vircell S.L., Grenade, Spain http://www.vircell.com	2, 3, 4
4. Plateia TOXO IgG, IgM	Whole tachyzoites	Bio-Rad, Marnes-la-Coquette, France http://www.bio-rad.com	2, 3, 4
5. Human® Toxo-IgG	Soluble	Human Biochemica und Diagnostica mbH, Wiesbaden, Germany http://www.human.de/de	2
**MEIA Microparticle Enzyme Immunoassay,** AxSYM Toxo IgG, IgM	Soluble	Abbott Laboratories, Illinois, USA http://www.abbott.com	2, 3, 4

### Review methodology

Using the key terms “Toxoplasma [and] Colombia” and “Toxoplasmosis [and] Colombia” to search publications from 1944 to 2014, we queried PubMed, Medline, SciELO and Google Scholar. National and international scientific journals were systematically reviewed and originals of all papers were consulted. After excluding summaries of conference reports, 90 publications met our selection criteria, including original articles (64), clinical trials (5), case reports (19) and reviews (2).

### Toxoplasmosis in humans

#### Seroprevalence and correlates of infection

**Figure 1:**
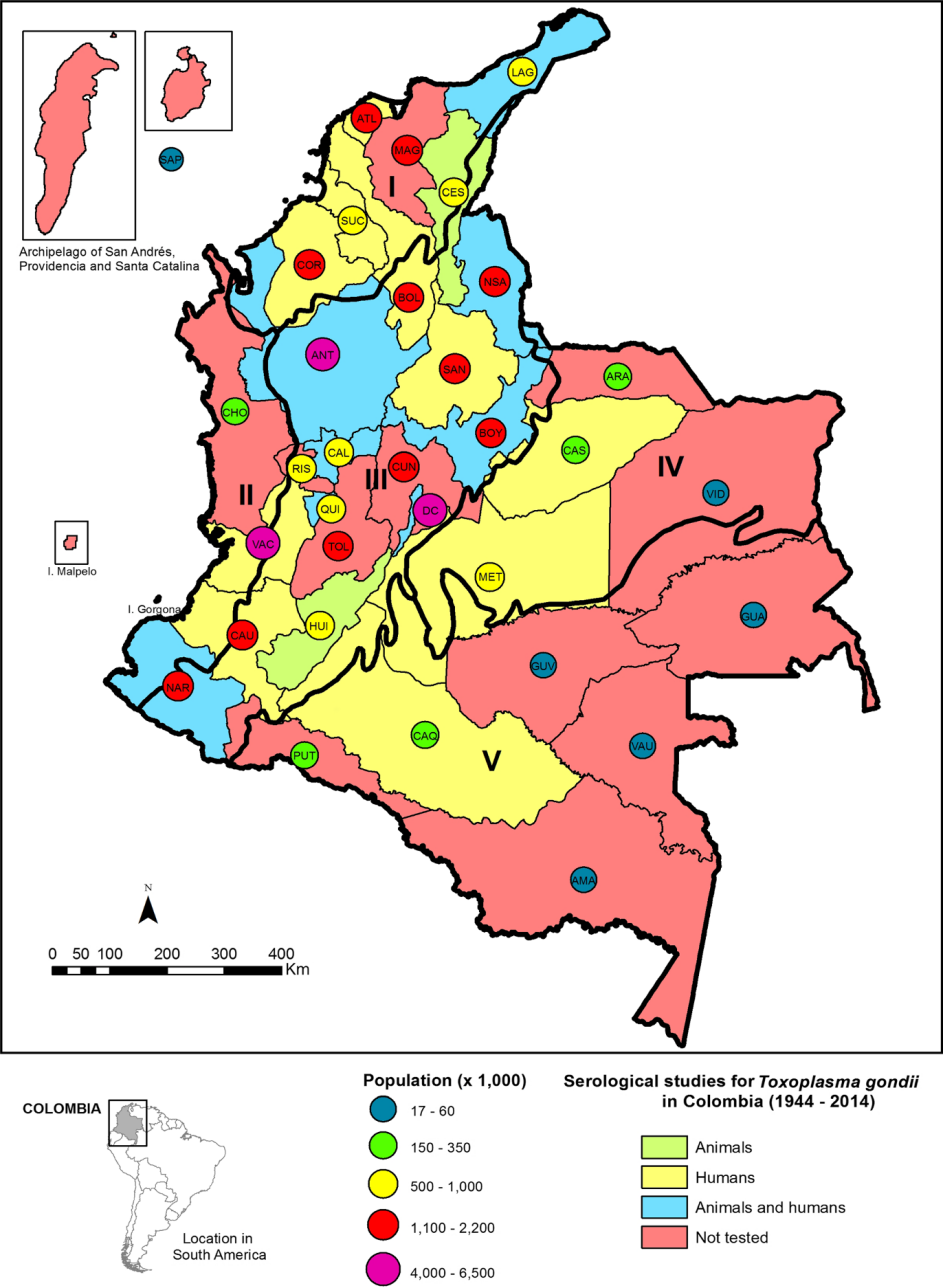
**Serological studies of** ***T. gondii*** **infection in humans and animals in Colombia (1944–2014).** On the map are indicated the natural regions and states of Colombia. **I Caribbean Region**: Archipelago of San Andrés and Providencia (SAP), La Guajira (LAG), Atlántico (ATL), Magdalena (MAG), Cesar (CES), Sucre (SUC), Bolívar (BOL), Córdoba (COR); **II Pacific Region**: Chocó (CHO), Valle (VAC), Cauca (CAU), Nariño (NAR); **III Andean Region**: Bogotá D.C (D.C), Norte de Santander (NSA), Antioquia (ANT), Santander (SAN), Risaralda (RIS), Caldas (CAL), Cundinamarca (CUN), Boyacá (BOY), Quindío (QUI), Tolima (TOL), Huila (HUI); **IV Orinoquía Region**: Arauca (ARA), Casanare (CAS), Vichada (VID), Meta (MET) and **V Amazon Region**: Putumayo (PUT), Caquetá (CAQ), Guaviare (GUV), Guainía (GUA), Vaupés (VAU), Amazonas (AMA).

Data are summarized in Tables 2, 3 and 4 and Figure 1.

**Table 2:** **Studies of seroprevalence of** ***T. gondii*** **in human populations conducted in Colombia**

Year	Population studied	Area on the map	No. tested	Test (cut-off)	No. positive (%)	Reference
**Low risk groups**						
1956	Indigenous Guambianos	CAU	297	SF (16)	88(29.63)	[11]
1956	Healthy patients	D.C, BOL, ATL, CAL, ANT	36	SF (NR)	22(61.11)	[14]
1959	Healthy individuals	D.C	205	SF (NR)	47(22.93)	[14]
	Mental patients		111		23(20.72)	
	Patients with diverse ailments		38		15(39.47)	
1968	Blood donors	ANT	184	SF (2)	98(53.26)	[15]
1969	Patients from Tunja Hospital	BOY	171	SF (8)	41(24.00)	[16]
	Soldiers	BOY	254		134(52.76)	
1974	Soldiers from several regions		1771	SF (16)	886(50.02)	[12]
1976	Pregnant women	ANT	120	SF (NR)	10(8.33)	[9]
1980	Pregnant women, nationwide survey		414	IFAT (16)	26(63.04)	[17]
1992	Pregnant women (National Institute of Health)	D.C	1000	IFAT (16)	590(59.00)	[18]
1993	Pregnant women	QUI	1617	IFAT (16)	1024(63.33)	[19]
1996	Pregnant women	CAS	51	IFAT(16)	37(72.55)	[20]
	Women of reproductive age		327		253(77.37)	
1997	Pregnant women	QUI	937	IFAT (16)	569(60.73)	[21]
1998	Pregnant women	D.C	637	IFAT (16)	299(46.94)	[22]
2003	Individuals without contact with dogs	CAL	300	IFAT (64)	132(44.00)	[23]
	Dog owners		306		99(32.35)	
2005	Pregnant women	MET	300	ELISA (>10 UI/ml)	158(52.67)	[24]
2005	Pregnant women	VAC	955	MEIA (NR)	(45.76)	[25]
2007	University students without ocular lesion	QUI	21	ELISA (>10 UI/ml)	13(61.91)	[26]
	University students with ocular lesion		12		9(75.00)	
2008	Group volunteers	Not Stated	140	ELISA (>1 UI)	74(52.86)	[27]
2009	National Institute of Health of Colombia	D:C	243	ELISA (>10 UI/ml)	137(56.38)	[28]
2012	Adult and child population	NAR	240	ELISA (>9 UI/ml)	108(45.00)	[29]
**High-risk groups**						
1981	Patients with clinical toxoplasmosis	VCA	44	IHAT (NR)	24(54.55)	[30]
1981	Handlers of slaughterhouses	ANT	169	IFAT (16)	45(26.63)	[31]
2001	HIV-positive patients	D.C	16	IFAT (128)	(93.75)	[32]
2003	Pregnant women with a history of abortion	SUC	100	ELFA (>8 UI/ml)	56(56.00)	[33]
2005	Patients with uveitis	D.C	25	ELISA (>9 UI/ml)	23(92.00)	[34]
2006	Veterinarians	MET	86	ELISA (>10 UI/ml)	36(44.19)	[35]
2007	HIV patients with cerebral toxoplasmosis	QUI	21	ELISA (>10 UI/ml)	16(76.19)	[36]
2008	Handlers in slaughterhouses	D.C,	82	ELFA (>8 UI/ml)	44(53.66)	[37]
		SAN	73		48(65.75)	
		ANT	72		45(62.50)	
		COR	80		67(83.75)	
		MET	93		83(89.25)	
2009	Soldiers operating in jungle	D.C	490	ELISA (NR)	394(80.41)	[38]
	Urban soldiers operating in Bogotá		501		226(4511)	
2011	Colombian newborn screening of *Toxoplasma*	QUI	1517	IC-ELISA (OD 8)	31(2.04)	[10]
		ATL	2901		2(0.07)	
		D.C	5398		12(0.22)	
		SAN	3036		7(0.23)	
		NSA	1124		0(0.00)	
		CAQ	510		9(1.76)	
		LAG	801		0(0.00)	

NR: not registered.

**Table 3:** **Correlates of seroprevalence of** ***T. gondii*** **in human population in Colombia**

Population studied (location)	No. tested	No. positive (%)	Correlates of infection	Reference
Healthy, mentally ill and other pathologies (Bogotá)	354	85 (24.0)	Patients with various disorders, >41 years old	[14]
Blood donors (Medellín)	184	98 (53.3)	16–30 years old	[15]
Pregnant women (Quindío)	1617	1024 (63.3)	Ownership and contact with cats, consumption of raw meat, 39 – 44 years old	[19]
Pregnant women (Villavicencio)	300	158 (52.5)	Contact with stray cats	[24]
Pregnant women (Cali)	955	437 (45.8)	30–39 years old, low socioeconomic level	[25]
Group volunteers Colombia – Italy	140	122 (50.8)	Age	[27]
Asymptomatic population (Manizales)	606	231 (38.1)	50–69 years old	[23]
Asymptomatic population (Pasto)	240	108 (45.0)	Adults, geographical differences, association with geohelminth infections	[29]
Handlers in slaughterhouses (Medellín)	169	45 (26.6)	Pig meat handlers 33–37 years old	[31]
Pregnant women with a history of abortion (Sincelejo)	100	56 (56.0)	Cat exposure	[33]
Handlers in slaughterhouses	400	287 (71.8)	Ingestion of raw meat, exposure to animals, contact with soil	[37]
Soldiers in operations in the Amazon rainforest (Bogotá)	1001	620 (61.9)	Geographical differences, untreated water consumption, consumption of wild meat	[38]
Colombian newborn screening of *Toxoplasma*	15.333	61 (0.39)	Rate of annual rainfall, geographical differences	[10]

**Table 4:** **Incidence of toxoplasmosis on seroconversion or acute markers rates in different Serological studies in Colombia (1994–2014)**

Population studied	Location	No. tested	Incidence (%)	Reference
Pregnant women	Medellin	120	8.3	[9]
Newborns	Bogotá	1320	1.4	[18]
Pregnant women	Armenia	896	1.3	[19]
Pregnant women	Quindío	933	1.6	[21]
Pregnant women	Sincelejo	100	2.0	[33]
Ophthalmic patients	Bogotá	25	12.0	[34]
Veterinarians	Villavicencio	86	4.6	[35]
Ophthalmic patients	Armenia	9	11.1	[26]
Pregnant women	Villavicencio	300	11.0	[24]
Pregnant women	Cali	995	2.8	[25]
Handlers in slaughterhouses	Bogotá	400	2.8	[37]
	Bucaramanga			
	Medellin			
	Monteria			
	Villavicencio			
Newborns (seven regions of the country)	Armenia	15333	0.5-6.2	[10]
	Barranquilla			
	Bogotá			
	Bucaramanga			
	Cúcuta			
	Florencia			
	Rioacha			
Asymptomatic population	Tuquerres	240	4.2	[29]
	Tumaco

There are several reports of *T. gondii* seroprevalence in the general human population, most of them based on convenience samples. A national study, using the indirect fluorescent antibody test (IFAT, cut-off 1:16), found an overall prevalence of 47.1% (4,304/9,139), with similar proportions in men (47.9%) and women (46.3%) and an increased risk of infection among pregnant women. They stated that seropositivity in 0–9 year old children was 32% (604 of 1,890) with similar prevalence in males (32.6%) and females (31.9%) [17]. The data in this report are difficult to interpret because, in most instances, only percentages of seropositivity are given without the number of subjects studied; there is a need for a new updated, population based study.

Data collected with respect to correlates of infection are also summarized in Table 3.

Handlers in slaughterhouses in Colombia were characterized as an occupational risk group [31, 37, 39], whereas this association was not established in Villavicencio veterinarians [35]. The presence of cats, geographical differences [17], age, environmental exposure, co-infection with *Ascaris lumbricoides* [29], sociocultural characteristics [27], educational level [40], untreated drinking water and ingestion of meat from wild animals [38] are epidemiological factors that have been associated with human toxoplasmosis in Colombia. Unrelated factors included physical condition [14], gender, consumption of undercooked pork [23], socio-demographic conditions [40] and altitude [12].

Various mathematical models of transmission dynamics of *T. gondii* in Colombia suggest a synergy between endemic levels of infection between cats and humans [41]. Consequently, the control of the feral feline population would have a significant effect on parasite dispersion by reducing environmental contamination by oocysts [42]. However, controlling the feral cat population does not interrupt *T. gondii* propagation [43] because of the proximate relationship between inoculum and infection [44], which is increased by the dispersion of *T. gondii* oocysts in water, which can even reach locales without a definitive host [45].

### Post-natally acquired infections

Little is known of clinical toxoplasmosis in the general population. Toxoplasmosis associated pneumonia was reported in a 26 year old [46], and nephritis in a 15 year old male [47].

Restrepo [48] mentioned a foodborne outbreak of toxoplasmosis. Lymphadenopathy and fever were observed in 11 of the 30 persons who participated in a barbecue where pork was the main food offered in 2005 in Jericó, Antioquia. Affected people became sick 10 to 15 days after the party and they were stated to have IgM *T. gondii* antibodies, but no other details were given. Thus, critical evidence concerning diagnosis is missing and it is unfortunate that these findings were mentioned in passing.

A waterborne outbreak of toxoplasmosis was reported in 18 individuals, 24 to 33 year old male Colombian soldiers deployed in a remote area in La Macarena, Meta. All patients had cervical lymphadenopathy, one had myocarditis, one had pneumonia, and one had diarrhea. All patients had high (>1024) IgM and IgG *T. gondii* antibodies. They were hospitalized, treated with pyrimethamine, sulfadoxine, and clindamycin, and all recovered. Drinking water contaminated with oocysts was thought to be the source of infection [49].

A well planned epidemiological investigation revealed that 80% of 501 Colombian soldiers operating in a jungle were seropositive to *T. gondii* and four (0.8%) had chorio-retinal lesions compared with 45% seropostivity in 501 soldiers deployed in urban Bogota, and only one (0.19%) had chorioretinitis. Drinking water was considered to be the source of higher seropositivity in jungle deployed soldiers [38].

### Pregnancy and congenital disease

Initial studies concerning acquired toxoplasmosis during pregnancy reported rates of 1.3% to 8.4% in different regions of Colombia (Table 4). Regardless of geographical region, the proportions were similar in studies based on double increase of IgG levels (by ELISA or IFAT technique) or with specific detection of IgM and IgA antibodies (Tables 2 and 4).

The national multicentric study revealed that the incidence of congenital toxoplasmosis was not homogenous, with significant variations between regions as well as a strong association among high mean annual rainfall (3,840-2,500 mm/year) and frequency of toxoplasmosis in women during pregnancy (3-6%). In addition, a mean of congenital infection was estimated for Colombia as one of 1,000 newborns (15 confirmed cases between 15,000 children studied); hence with a population of 550,000 newborn per year in the nationwide (Table 5), it is expected about 550 infected newborns per year [10].

**Table 5:** **Clinical toxoplasmosis in congenitally-infected children in Colombia**

Reference	Location	Type of sample	No. tested	Infected (%)	Sex (%)	Deaths (%)	Neurological symptoms and signs (%)	Ocular symptoms and signs (%)	Hepato-splenomegaly (%)	Prematurity (%)
1981 [30]	Cali	Referred cases (1955–1975), confirmed by necropsy (21 cases) or with serological studies	44	NA	Male 25 (56.8)	21 (47.7)	Microcephaly and cerebral calcifications: 30 (68.1)	25 (56.8)	27 (62.5)	8/16 (50.0)
1983 [50]	Medellin	Referred cases confirmed by IFAT test	27	NA	Male 17 (63.0)	NR	Microcephaly: 20 (74.0) Neurological psicomotor deficit: 22 (81.5) Electroencephalogram changes: 8/14 (57.0)	Strabismus: 9 (33.0) Cataract: 1 (3.7) Chorioretinitis 11(40.7)	8 (29.0)	5 (18.5)
1997 [21]	Quindío	Children prenatal screening	15	1/15 (6.7)	Female	NR	NR	Chorioretinitis (6.7)	NR	NR
2000 [51]	Bogota Armenia	Referred cases confirmed by IFAT and ISAGA test	27	NA	NR	1 (3.7)	Calcification, hydrocephaly or microcephaly: 12 (44.4)	Chorioretinitis 11 (40.7)	16 (59.0)	5 (18.5)
2005 [52]	Armenia	Children prenatal or newborn screening	26 (17 by screening and 11 symptomatic)	NA	NR	2 screened <6 mo. of age (11.7%) 1 of 11 symptomatic (9%).	17 screened 4 of 11 symptomatic (36%)	13 screened 4 symptomatic (30%)	26 screened 7 symptomatic (26.9%)	NR
2006 [53]	Quindío	Newborn screening in referral hospital	200	1/200 (0.5)	Female	1 (Respiratory distress syndrome)	NR	NR	NR	NR
2007 [54]	Quindío	Newborn screening in community hospitals	322	2/322 (0.5)	NR	NR	NR	NR	NR	NR
2011 [10]	Armenia Barranquilla Riohacha Cucuta Bogota BucaramangaFlorencia	National multicentric newborn screening	15,333	218 (1.4) by criteria for confirmation assays (109; 50% by confirmatory assays; 15 (13.7%) by congenital infection and 3/15 newborns with prenatal treatment	NR	3/15 (20.0)	4/15 (26.6) calcifications; 1/15 (6.6) hydrocephaly	3/15 (20.0) chorioretinitis	1/15 (6.6) splenomegaly	1/15 (6.6)

NA: not applicable; NR: not registered.

Colombian gynecologists currently use molecular methods to determine the fetal transmission of *T. gondii*. DNA of *T. gondii* was detected in 10.1% of amniotic fluid by amplification of the B1 gene in positive samples of mothers with serological criteria for acute toxoplasmosis in Bogotá [55]. PCR on maternal blood samples is not a confirmatory test of fetal infection; nevertheless, a PCR-B1 assay described a positivity of 12% in blood samples of positive pregnant women from Sincelejo [56]. Moreover, a serologic test (ELISA ELFA) showed in the same location seroconversion of 2% during gestational control and neonatal mortality [33] and exposed the burden of congenital toxoplasmosis in the Caribbean region.

As mentioned in the introduction, the severity of congenital toxoplasmosis has been recognized in Colombia since 1949, when the first case was diagnosed and reported in 1951 [8, 30, 51–53]. Ophthalmic complications also have been reported as strabismus and bilateral chorioretinal scars [57], hydranencephaly [58], vitreous hyper-echogenicity, severe hydrocephalus [59] and neuro-ophthalmic infection [60].

Because until now there was no planned *T. gondii* screening program to follow pregnant women and infected children, the information on clinical toxoplasmosis in children in Colombia is fragmentary. Here, we attempted to summarize available information in Table 5. Although these reports were based on sporadic cases from Colombian referral centers, it is likely that the apparent clinical severity is associated with geographical differences. Indeed, the multicenter study [61], with 25 cohorts of infected mothers from Europe, North America, and South America, provided unexpected results and concluded that ocular risk (47%) and intracranial lesions (53%) among Colombian children far exceeded that of European children (14% and 9% respectively).

There is no depository of *T. gondii* isolates from Colombia. One *T. gondii* isolate (designated CIBMUQ/HDC) from blood of a congenitally infected child in Quindío, Armenia has been deposited in the French National Collection [62]. The infected child was born to a 13-year old mother when she was in the 33 week of gestation. The child had hepato-splenomegaly and icterus. Viable *T. gondii* was isolated from the peripheral blood buffy coat of the child by bioassay in mice and cell culture. The strain was mouse-virulent and genotype I (see section on genotyping).

In Colombia, risk factors associated with congenital infection are contact with cats [24, 33], spatial dispersion of *T. gondii* by domestic cats [44], eating undercooked meat or ingesting untreated water [63], living in households in marginal areas, socio-economic status [25] and geographic differences and rainfall [10] (Table 3).

### Ocular disease

In Cali, the Institute for Deaf and Blind Children lists toxoplasmic chorioretinitis as the second leading cause of congenital blindness [64], and it is the third leading cause according to the ophthalmologic evaluation of infants under 16 years of age (19/127), affecting a higher proportion of girls between six months and six years of age (63.2%) [65], and three cases of ocular toxoplasmosis occur per 100,000 inhabitants in Quindío [66].

Chorioretinal lesions were diagnosed in patients from rural and urban areas of Bogotá [34], and in 6% (12/200) of the student population of the University of Quindío [26]. Posterior uveitis (67.2%), panuveitis (46.6%) and unilateral (79.0%) lesions were observed in 39.5% (109/276) in two Colombian referral centers in Quindío and Bogotá [67]. Active lesions (45.0%) and recurrent retinochoroiditis (59.3%) are responsible for decreased visual acuity in 60.5% of cases with remarkable visual dysfunction in bilateral condition [68].

In recurrent ocular toxoplasmosis, atypical uveitis is the most common complication [69]. Episodes of recurrence can occur at approximately 11 year intervals and are associated with the presentation of inactive chorioretinal lesions and antibiotic therapy without accompanying steroid treatment [70].

### Immunocompromised patients

An estimated 7,000 to 10,000 new cases of toxoplasmosis in positive HIV patients occur annually in Colombia [71], with cerebral toxoplasmosis (CT) as the main complication. *Toxoplasma* seropositivity was recorded in patients from Bogotá (15/16 cases) [32] and in 54 cases from Cúcuta, with a post-diagnosis survival of 50% [72]. In a study of 821 autopsies in one hospital in Santander from 2004–2007, *T. gondii* –associated lesions and parasites were found in 17 (28.3%) of 60 cases of HIV infected persons [73] and a single case report in Huila [74]. Brain tomography, IgG antibody detection, detection of *T. gondii* DNA in peripheral blood are considered effective for the diagnosis of CT [36, 75].

CT shows effects on consciousness, neural disorders and orientation [36] and complications may occur, including chorioretinitis with opacity of the optic nerve [76], and infection of the spinal cord with involvement of lower limb motor function; *T. gondii* tachyzoites were identified immunohistochemically in biopsy of the thoracic spinal cord [77].

### Toxoplasmosis in animals

#### Clinical

Four months after a flock of Blackface sheep imported from Great Britain disembarked in Colombia, premature births and abortions occurred within the first 48 hours after birth; bioassays in mice and the histopathological analysis of fetal and placental products identified *T. gondii*, and 44 females tested positive by indirect hemagglutination tests [78]. It was speculated that ewes might have become infected with *T. gondii* during prolonged quarantine, and stress of transportation in Great Britain during transit to Colombia might have caused abortion.

As part of rabies surveillance, brains of animals suspected to have rabies from 1967–1972 were examined for Negri bodies and by mouse inoculation: *T. gondii* was found in 2 of 235 cats, 8 of 772 dogs, 3 of 93 rats and 1 of 1 guinea pig [79]. The clinical significance of these findings is uncertain.

We are not aware of any other reports of clinical toxoplasmosis in Colombia.

### Serological and parasitological prevalence

Data are summarized in Tables 6, 7 and Figure 1.

**Table 6:** **Seroprevalence studies of** ***T. gondii*** **in domestic animals in Colombia**

Year	Area on the map	Species	Number	Serologic test (cut-off)	No. positive (%)	Reference
1965	D.C	Dogs	1000	SF (8)	157 (15.7)	[80]
2003	CAL		306	IFAT (32)	124(40.5)	[23]
2007	D.C		309	MAT (20)	52(16.8)	[81]
1970	ANT	Cats	181	SF(8)	112(61.9)	[15]
2006	QUI D.C		25137	MAT (20)	21(84.0) 31(21.3)	[82]
2006	CAL	Horses	777	IFAT (32)	164(21.1)	[83]
1981	ANT	Pigs	368	HIA (64)	111(30.2)	[84]
2006	CAL		797	IFAT (32)	122(15.3)	[83]
1981	ANT	Cattle	371	HIA (64)	90(24.3)	[84]
1981	ANT		361	IFAT (16)	108(29.9)	[31]
2006	CAL		397	IFAT (32)	140(35.3)	[83]
2005	QUI	Backyard chickens	77	MAT (20)	25(32.4)	[85]
2006	CAL		955	IFAT (64)	149(15.6)	[83]
1978	BOY	Sheep	1141	IHAT (16)	724(63.5)	[86]
	CES		46		30(65.2)	
	LAG		145		86(59.3)	
	HUI		96		34(35.4)	
	NAR		130		59(45.4)	
	NSA		97		25(25.8)

**Table 7:** **Isolation of viable** ***T. gondii*** **from tissues of animals in Colombia**

Host	Region	Type	No. bioassayed	Tissues bioassayed	No. positive	Mouse virulence^a^	***T. gondii***isolate designation	Reference
**Chicken**	Quindío	Free range	72	Heart, brain	23	16	TgCkCo1-23	[85, 87]
**Cat**	Armenia Bogotá	Unwanted	116	Heart, brain	15	9	TgCtCo1-15	[82, 87]
**Dog**	Bogotá	Unwanted	37	Heart, brain, tongue	20	4	TgDgCo1-20	[81]

^a^100% mortality in outbred mice.

### Cats

Seroprevalence in cats deserves special attention because of the epidemiological importance as definite host of *T. gondii*. Jewell *et al.* [15] surveyed people and pet cats from Medellin; 112 (62%) of 181 cats had dye test antibodies with titers of 8 in 16, 32 in 34, 128 in 38, and 512 in 24 cats. Montoya *et al.* [88] reported IFAT antibodies in 25 of 28 cats from the city of Armenia, but little else was said of the cats surveyed nor of the IFAT titer. Dubey *et al.* [82] reported *T. gondii* antibodies in 52 (30.5%) of 170 cats with titers of 1:20 in 10, 1:40 in 7, 1:80 in 4, 1:160 in 8, 1:320 in 6, and 1:640 in 17. Thus, most of the cats had high titers; 21 (84%) of 25 cats from Armenia were seropositive compared with 31 (21.3%) from Bogota. Viable *T. gondii* was isolated from tissues of 15 of 42 cats with MAT titers of 1:40, but not from any of the 90 cats with titers of 1:20 or lower.

As of yet, viable *T. gondii* oocysts have not been demonstrated in cat feces in Colombia. Oocysts were not found by microscopic examination or by bioassay of feces of the 170 cats from Armenia and Bogota [82]. Montoya *et al.* [88] found *T. gondii*-like oocysts in 18 of 28 cats from Armenia, but there is no evidence to judge the validity of the findings. Herrera *et al.* [89] said that they isolated *T. gondii* from the feces of a cat but there is no other information about the cat and method of isolation. With respect to the demonstration of *T. gondii* oocysts, bioassay is essential because there are other *T. gondii*-like parasites in cat feces [13].

### Food animals as sources of infection

Poultry, beef, pork and mutton are the most important sources of meat consumed by humans in Colombia. Only limited information is available concerning the prevalence of *T. gondii* in food animals (Table 6). Two surveys that were performed more than 30 years ago indicated a high prevalence of antibodies in cattle in Medellin (Table 6). A relatively recent study reported 140 (35.3%) of 397 cattle from Manizales were seropositive using a cut-off of 1:32 in the IFAT [83]. Cattle are considered a poor host for *T. gondii* and it is extremely rare to isolate viable *T. gondii* from beef [13]. Moreover, several serological tests, including the IFAT and IHAT used give a false positive unless the cut-off is high [13]. Thus, the role of beef in the epidemiology of *T. gondii* is uncertain.

A high prevalence (57.9%) of antibodies was recorded in sheep in six regions of the country (Table 6), but this study was done more than 35 years ago [86]. *T. gondii* infections in pigs had been recorded in 1979 and 1981 but these are also old studies [90, 91]. More recently, *T. gondii* antibodies were found in 15.3% of 797 > 8 months old pigs from Caldas [83]. The same study reported *T. gondii* antibodies in 15.6% of 955 chickens. There is no information with respect to *T. gondii* infection in goats used for meat.

Currently, there is a great public interest in food safety and the presence of viable *T. gondii* in meat. Serological surveys from slaughtered animals and the detection of parasite DNA in meat do not provide a true assessment of risk to humans because conditions for storage and treatment of meat from the time of slaughter and consumption affect the viability of parasites. Therefore, studies are needed to detect the presence of viable *T. gondii* in meat from retail meat markets. Lora *et al.* [92] found *T. gondii* DNA by PCR in 95 (52.7%) of 180 (60 samples each) meat samples (42 pork, 29 beef, 24 chicken meat). This is an alarming rate of contamination of meat samples from retail stores and needs confirmation.

### Contamination of the environment with T. gondii

There are no specific data on the contamination of the soil and the environment with *T. gondii* oocysts in Colombia. However, the high seroprevalence of *T. gondii* in cats suggests that the environment is likely to be contaminated, because cats that are seropositive have shed oocysts [13]. Although *T. gondii* oocysts are shed for only 1–2 weeks in the life of the cat, millions of oocysts can be shed and they can survive outdoors for months.

Seroprevalence of *T. gondii* in free range chickens (small farms) is more indicative of soil contamination, because chickens feed from the ground, than as a food source for the main population. Seroprevalence in 77 free range chickens from 9 farms was 32.4%, using a MAT titer of 20, and viable *T. gondii* was isolated from 15 of the seropositive chickens [85]. Seropositive chickens were found on all properties, indicating widespread soil contamination in rural Quindío, Colombia.

The high seroprevalence of *T. gondii* in herbivores (Table 6) also indicates that the rural environment is also contaminated with oocysts. For example, the ingestion of oocysts is the main mode of transmission of *T. gondii* in sheep.

Dogs have been used as sentinel animals for estimating *T. gondii* infection in the environment because of their close contact with humans. Actually, dogs were found to be risk factor for *T. gondii* infection in people in Panama [93]. Dogs are known to eat cat feces and roll over in cat feces. Thus, their fur becomes contaminated with oocysts and children can become infected by petting infected dogs. In dogs, the prevalence for *T. gondii* was recorded in Bogota and Manizales; gender, age, race and type of feeding showed no significant correlation [23, 81, 91].

Recently, attention has been drawn to the prevalence of *T. gondii* in bats and epidemics of bat mortality. Most bat species are insectivores and live in caves. Thus, infection in these bats indicates contamination of caves by oocysts. In this respect, two of 38 *Artibeus lituratus* bats captured in Tibú, Santander had dye test antibodies [16].

### Genetic characterization of T. gondii strains from Colombia

Information on genetic typing is summarized here. The quality of DNA is important for genetic typing and complete data can be obtained only from DNA extracted from large numbers of viable parasites, usually cell or mouse-cultured organisms. More limited information can be obtained on DNA extracted directly from tissues of asymptomatic animals. Humans become infected with *T. gondii* mostly by consumption of uncooked meat or the oocysts. Therefore, information on genotypes of isolates from animals, especially cats, is relevant to human infections.

Different methods have been used to type the isolates. Earlier information was obtained using serotyping [94] and polymerase chain reaction-restriction fragment length polymorphism (PCR-RFLP) with only SAG2 marker [85, 95]. Gallego *et al.* [95] detected *T. gondii* DNA in 50 of 146 samples from humans and animals but did not identify the samples of each species tested. Of the 50 PCR-positive samples, they characterized 33 samples using the SAG2 gene; 14 human samples (6 congenital infection, 3 HIV patients, 1 ocular toxoplasmosis, 3 pregnant women, 1 case of myositis), 2 *Myarchus cephalotes*, 15 cats (8 brains, 6 hearts, 1 fecal sample), 1 *Didelphis marsupialis*, and 1 guinea pig. It is really unfortunate that details of hosts and samples were not stated. By using the SAG2 gene, 31 of 33 samples were genotype SAG2 type I. Now we know that SAG2 typing in strains from South America is insufficient because of the polymorphic nature of the strains in this region need multilocus analysis. One of these strains (CIBMUQ/HDC) from the congenitally infected child was subsequently typed using six microsatellite markers (*TUB2, TgM-A, W35487, BM189462, BM175053, N82375*) and it was found to be type I; it is mouse virulent [62].

We have recently genotyped 53 isolates of *T. gondii* from cats, dogs, and chickens from Colombia using 11 RFLP markers (Tables 7, 8). Nineteen genotypes were obtained, out of which only two were clonal and both of these were type I (Table 7). Type I strains are extremely rare worldwide. Thus, finding three Type I strains out of 54 isolates from Colombia suggests the need for further study.

**Table 8:** **Genotypes of** ***T. gondii*** **from cats, dogs, chickens from Colombia based on 11 RFLP markers** [87]

Toxo DB type^a^	No. of isolates	Designation
9, (Chinese1)	1	TgDgCo4
10 (Type I)	2	TgCtCo 2, 7
14	6	TgDgCo9,12,15,18; TgCkCo2;TgCtco14
18	3	TgCtCo 12, 13; TgDgCo3
23	1	TgDgCo19
28	2	TgCtCo 1; TgCkCo5
29	5	TgDgCo1,2,10,20; TgCkCo20
38	13	TgCkCo6,8,10,12,13,15,21,23,24; TgCtCo 4,10,11; TgDgCo17
40	1	TgCtCo5x
44	3	TgDgCo5,6,11
46	3	TgDgCo8,14,16
61	2	TgCtCo 5,6
62	2	TgCtCo3,9
79	1	TgDgCo13
101	1	TgCtCo15
122	1	TgDgCo7
128	1	TgCtCo8
178	1	TgCkCo4
179	2	TgCkCo17,22
188	1	TgCkCo9

Before the discovery of the methods to genotype, *T. gondii* strains were grouped as virulent or avirulent for mice. Howe and Sibley [96] proposed that *T. gondii* isolates can be grouped in to three types (I, II, III) based on RFLP typing, and that most strains were clonal. Additionally, Type I strains were 100% lethal for mice, whereas Types II and III were less pathogenic. Recent studies have shown that *T. gondii* isolates are genetically diverse, particularly those from Brazil and Colombia [4]. Now more than 200 genotypes of *T. gondii* are known; most of these are from South America, and there is an International *Toxoplasma* data base (http://www.toxodb.org) to record the different genotypes. Here, we have used the ToxoDB to record genotypes from Colombia (Table 8).

Additionally, *T. gondii* isolates from Colombia and Brazil were phenotypically different; 80% of *T. gondii* strains from Colombia were 100% lethal to outbred mice (Table 7), but only two were not Type I.

Currently, there is great scientific interest in finding the molecular basis of pathogenicity of *T. gondii* isolates. Now several genes, including ROP18, are found associated with virulence, but mouse virulence may not apply to all hosts [97].

As stated earlier, most of the *T. gondii* virulence studies have focused on infections in mice. Recently, scientists in Colombia have attempted to correlate severity of clinical toxoplasmosis in patients with genetic make of the strain and host responses (cytokine production), and found that the virulent allele of *T. gondii* ROP18 in ocular toxoplasmosis was correlated with severe ocular inflammatory response [98]. This study additionally found that the cytokine profile in Colombian patients with ocular toxoplasmosis was deviated to a Th2 profile [99]; instead, French patients had a Th1 preferential response [100]. Altogether, these results indicate that some Colombian strains cause more severe ocular toxoplasmosis due to an inhibition of the protective effect of IFN-γ. These findings afford new research avenues to revert the Th2 deviated immune response in patients with severe forms of ocular toxoplasmosis.

## Conclusions and perspective

From the information summarized here, it is evident that the toxoplasmosis is a major public health problem and more than half of the women of child bearing age are seronegative for *T. gondii* and at risk of exposure to *Toxoplasma* during pregnancy and congenital transmission to their fetuses. Additionally, the clinical disease in congenitally infected children is more severe in Colombia than in Europe. It is tempting to speculate that severity of toxoplasmosis in children is, in part, related to unusual genetic types of *T. gondii* circulating in Brazil and Colombia. However, only one strain of *T. gondii* from a congenitally infected child from Colombia has been genotyped. Although most studies on toxoplasmosis in Colombia are limited to one region, studies during prenatal and newborn programs with adequate follow up of children are needed in order to ascertain the extent of clinical damage to children and to correlate with strain genetic typing. Evaluation of the current official, evidence-based guidelines will be needed to evaluate the impact on reducing the burden and sequelae of congenital infection. Little is known of sources of infection with *T. gondii* in humans and animals in Colombia. The level of contamination of the environment by oocysts and the percentage of food animals infected with viable *T. gondii* is also unknown. Colombia has vast rural areas and diverse wildlife. Virtually, nothing is known of the role of wildlife in the circulation of *T. gondii*.
